# Instrumental Activities of Daily Living Difficulties Predict Cognitive Decline in Older Puerto Rican Adults

**DOI:** 10.1093/geroni/igab046.914

**Published:** 2021-12-17

**Authors:** Caitlin Pope, Tyler Bell, Brian Downer, Sadaf Milani, Lauren Roach, Michael Crowe

**Affiliations:** 1 University of Kentucky, Lexington, Kentucky, United States; 2 University of California San Diego, La Jolla, California, United States; 3 University of Texas Medical Branch, Galveston, Texas, United States; 4 University of Alabama at Birmingham, Birmingham, Alabama, United States

## Abstract

Given the hypothesized bidirectional association between functional and cognitive decline, further characterization of the temporal association between the two is needed, especially in Latinx samples as they are the most rapidly growing demographic in the United States and at greater risk for Alzheimer’s disease. This study assessed bidirectional associations between instrumental activities of daily living (IADL) difficulty and cognition in older Puerto Rican adults. Participants included 2,840 community-dwelling adults (60+ years) without cognitive impairment who completed baseline and a four-year follow-up in the Puerto Rican Elderly: Health Conditions (PREHCO) project. At each wave, cognition (using the Mini-Mental Cabán) and self-reported IADL difficulty (a sum score of 10 everyday tasks) were measured. Covariates included age, gender, education, comorbidities, and depressive symptoms. Bidirectional associations were tested using a path model with concurrent and cross-lagged paths between cognition and IADL difficulty adjusting for covariates. Lower baseline cognition related to more baseline IADL difficulty (B=-0.08, SE=0.02, p<.001). Cognitive decline at follow-up related to greater IADL difficulty at follow-up (B=-0.06, SE=0.02, p=.012). Looking at cross-lagged associations, greater baseline IADL difficulty associated with more cognitive decline at follow-up (B=-0.10, SE=0.04, p=.012). However, baseline cognition was not significantly associated with change in IADL difficulty at follow-up (B=-0.003, SE=0.02, p=.869). Findings support the growing body of literature that IADL difficulties can predict future cognitive decline in samples of community-dwelling older adults. More research into both functional and cognitive decline in Latinx samples will provide a more generalizable view of aging.

